# Cross-sectionally Calculated Metabolic Aging Does Not Relate to Longitudinal Metabolic Changes—Support for Stratified Aging Models

**DOI:** 10.1210/clinem/dgad032

**Published:** 2023-01-20

**Authors:** Mika Ala-Korpela, Terho Lehtimäki, Mika Kähönen, Jorma Viikari, Markus Perola, Veikko Salomaa, Johannes Kettunen, Olli T Raitakari, Ville-Petteri Mäkinen

**Affiliations:** Systems Epidemiology, Faculty of Medicine, Center for Life Course Health Research, University of Oulu, Oulu 90014, Finland; Biocenter Oulu, University of Oulu, Oulu 90014, Finland; Faculty of Health Sciences, NMR Metabolomics Laboratory, School of Pharmacy, University of Eastern Finland, Kuopio 90014, Finland; Department of Clinical Chemistry, Faculty of Medicine and Health Technology, Fimlab Laboratories, and Finnish Cardiovascular Research Center Tampere, Tampere University, Tampere 33100, Finland; Department of Clinical Physiology, Faculty of Medicine and Health Technology, Tampere University Hospital, and Finnish Cardiovascular Research Center Tampere, Tampere University, Tampere 33100, Finland; Department of Medicine, University of Turku, Turku 20520, Finland; Division of Medicine, Turku University Hospital, Turku 20520, Finland; Department of Public Health and Welfare, Finnish Institute for Health and Welfare, Helsinki 00271, Finland; Estonian Genome Center, University of Tartu, Tartu 51010, Estonia; Department of Public Health and Welfare, Finnish Institute for Health and Welfare, Helsinki 00271, Finland; Systems Epidemiology, Faculty of Medicine, Center for Life Course Health Research, University of Oulu, Oulu 90014, Finland; Biocenter Oulu, University of Oulu, Oulu 90014, Finland; Department of Public Health and Welfare, Finnish Institute for Health and Welfare, Helsinki 00271, Finland; Research Centre of Applied and Preventive Cardiovascular Medicine, University of Turku, Turku 20520, Finland; Department of Clinical Physiology and Nuclear Medicine, Turku University Hospital, Turku 20520, Finland; Centre for Population Health Research, University of Turku and Turku University Hospital, Turku 20520, Finland; Systems Epidemiology, Faculty of Medicine, Center for Life Course Health Research, University of Oulu, Oulu 90014, Finland; Computational and Systems Biology Program, Precision Medicine Theme, South Australian Health and Medical Research Institute, Adelaide, SA 5000, Australia; Australian Centre for Precision Health, University of South Australia, Adelaide, SA 5000, Australia

**Keywords:** metabolomics, metabolic aging, epidemiology, biological age, chronological age, stratified aging model, molecular clocks

## Abstract

**Context:**

Aging varies between individuals, with profound consequences for chronic diseases and longevity. One hypothesis to explain the diversity is a genetically regulated molecular clock that runs differently between individuals. Large human studies with long enough follow-up to test the hypothesis are rare due to practical challenges, but statistical models of aging are built as proxies for the molecular clock by comparing young and old individuals cross-sectionally. These models remain untested against longitudinal data.

**Objective:**

We applied novel methodology to test if cross-sectional modeling can distinguish slow vs accelerated aging in a human population.

**Methods:**

We trained a machine learning model to predict age from 153 clinical and cardiometabolic traits. The model was tested against longitudinal data from another cohort. The training data came from cross-sectional surveys of the Finnish population (*n* = 9708; ages 25-74 years). The validation data included 3 time points across 10 years in the Young Finns Study (YFS; *n* = 1009; ages 24-49 years). Predicted metabolic age in 2007 was compared against observed aging rate from the 2001 visit to the 2011 visit in the YFS dataset and correlation between predicted vs observed metabolic aging was determined.

**Results:**

The cross-sectional proxy failed to predict longitudinal observations (*R^2^* = 0.018%, *P* = 0.67).

**Conclusion:**

The finding is unexpected under the clock hypothesis that would produce a positive correlation between predicted and observed aging. Our results are better explained by a stratified model where aging rates per se are similar in adulthood but differences in starting points explain diverging metabolic fates.

Understanding individual human aging is an important topic for individual and public health (1, 2). The recent surge in metabolomics (3, 4) has resulted in large-scale studies on human populations and initiated hope—and hype (5)—regarding how these data would solve the raison d’être of molecular aging and provide clues to mitigate these processes. A cross-sectional survey of young and old people is the easiest to organize, but it yields the weakest epidemiological evidence; only longitudinal designs can provide well-grounded information on life course trajectories (6-10). So far, most metabolomics aging studies have been cross-sectional (11-15).

In the absence of longitudinal data, the concept of biological or metabolic age (Fig. 1, A) has been introduced to capture age-related shifts in metabolism and other cellular processes (15-22). Typically, a cross-sectional multivariate regression model of age is constructed with the metabolites as the regressors. The residuals of the model are then interpreted as the difference between an individual's chronological and metabolic age (ie, a positive residual implies accelerated metabolic aging). The caveats of cross-sectional modeling are well recognized (5, 16, 23) although longitudinal data collections come with their own caveats. Time series data are not free from epidemiological confounding or statistical fluctuations. In addition, samples collected years apart are likely to be influenced by collection protocols, storage effects, and changes in biochemical assay methodologies. For these very reasons, we recently introduced new statistical procedures to assess sample quality and to adjust for bias in longitudinal metabolomics data (24).

**Figure 1.**
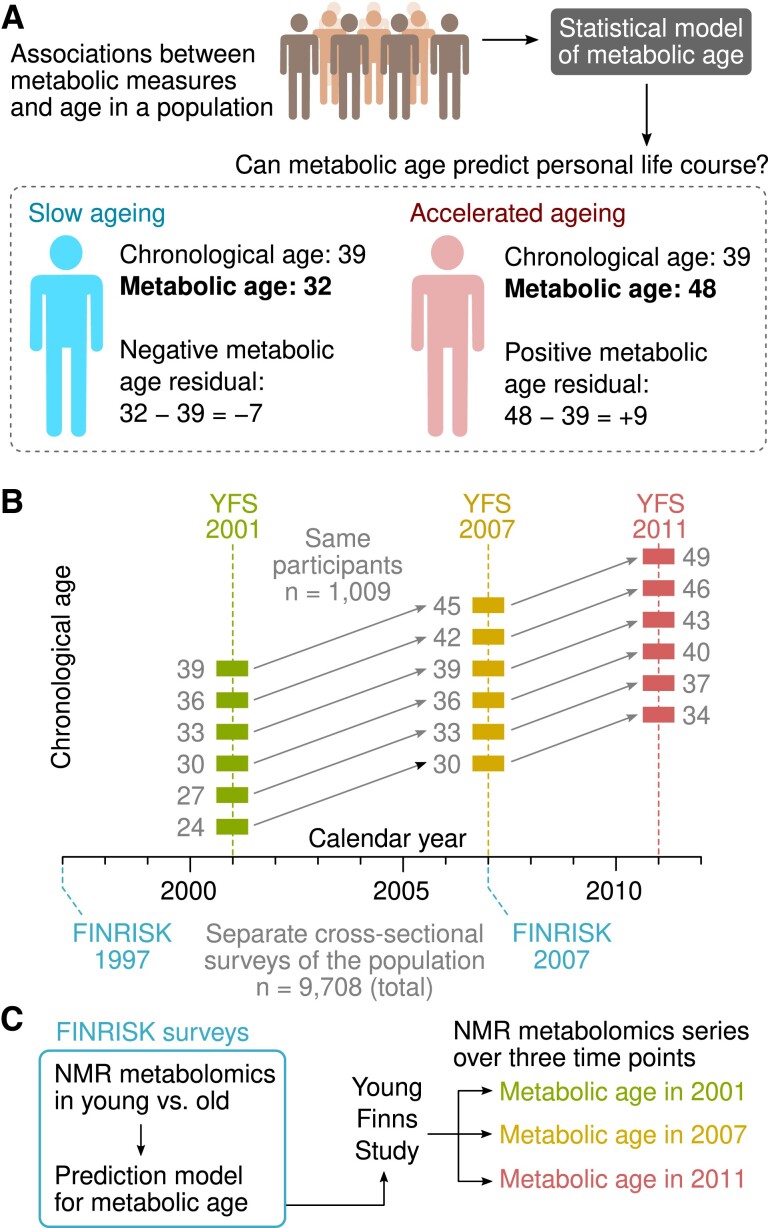
Overview of the study design and datasets. (A) The concept of biological or metabolic age has been introduced as a proxy measure of human aging processes. First, quantitative biological data, such as metabolomics, are collected in a cross-sectional cohort. Next, a statistical model is built to predict an individual's chronological age from the biological data. Lastly, the deviation of the model output from actual age (metabolic age residual) is interpreted as an indicator of the rate of biological aging. (B, C) We used a unique serum NMR metabolomics time-series resource based on the Cardiovascular Risk in Young Finns Study (YFS) that has been calibrated for longitudinal studies. For the first time it was possible to observe the rate of change in metabolic age directly and evaluate the usefulness of the cross-sectional metabolic age as an indicator of human aging processes.

In this study, we test the validity of the metabolic age concept by comparing cross-sectional predictions of accelerated aging (*n* = 9708 participants in the training set) against the observed rate of change across 3 consecutive time points in an independent longitudinal dataset (*n* = 1009 participants and 10 years of follow-up).

## Methods

FINRISK surveys are cross-sectional, population-based studies conducted every 5 years since 1972 to monitor the risk of chronic diseases (25). For each survey, a representative random sample was selected from 25- to 74-year-old inhabitants of different regions in Finland. The current study included eligible participants from FINRISK surveys conducted in 1997 and 2007. Data collection including clinical examination and serum samples were available for these 2 surveys. Serum samples were stored at −70 °C. Samples were semi-fasting: participants were asked not to eat 4 hours prior to giving blood. The median fasting time was 5 hours (interquartile range, 4-6 hours). Clinical and serum metabolomics data were available from 5304 participants (mean age 48 ± 13 years) in the 1997 survey and 4616 participants (mean age 52 ± 13 years) in the 2007 survey.

The Cardiovascular Risk in Young Finns Study (YFS) is a population-based prospective cohort study (26). It was conducted at 5 medical schools in Finland (Turku, Helsinki, Kuopio, Tampere, and Oulu), with the aim of studying the levels of cardiovascular risk factors in children and adolescents in different parts of the country. The baseline study in 1980 included 3596 children and adolescents aged between 3 and 18 years. Results from clinical examination and fasting samples were used in the present study. Clinical and serum metabolomics data were available from 3 visits; in 2001 (1239 women and 1007 men), 2007 (1186 women and 974 men) and 2011 (1112 women and 927 men).

### Clinical Biomarkers and Sample Quality

Glucose, insulin, triglycerides, low- and high-density lipoprotein cholesterol, C-reactive protein, and creatinine were assessed by standard clinical assays. To ensure the best possible estimates for age-associated changes in absolute concentrations, we employed a two-stage pre-processing protocol (24). First, we constructed multivariate regression models of biomarkers that were available both from metabolomics and clinical assays (glucose, triglycerides, total cholesterol, high-density lipoprotein cholesterol, and serum creatinine). Each nuclear magnetic resonance (NMR) measure was predicted from the combination of 6 clinical biomarkers and the model residuals were considered indicative of data consistency. The final quality score was defined as the standardized sum of residuals over the biomarkers. Hence samples with unusual and correlated residuals in multiple biomarkers were likely to get an extreme quality score. The cutoffs for acceptable deviation were set at the points where there was greater than 5% chance that the observed quality score was from the expected normal distribution. Consequently, 7.9% of samples in the YFS and FINRISK were excluded. In the final analyses there were thus 9708 participants in the cross-sectional training data set and 1009 participants in the independent test data set, for whom there were clinical and serum metabolomics data available in all the 3 YFS time points at 2001, 2007, and 2011.

### Calibration Between Visits

The second correction was aimed at bias between visits. We exploited longitudinal biomarker data in YFS to calibrate consecutive visits. We assumed a priori that subpopulations of the same sex, average age, and body mass have identical average metabolic profiles. This means that if subsets of individuals from 2 consecutive visits have identical average features, we would expect the subset averages of the biochemical data to be identical as well.

Given consecutive visits A and B in the YFS cohort, a subset of participants was selected from visit A and another mutually exclusive subset from visit B. The selection was optimized so that the subsets had matching age, sex (225 men and 225 women), and body mass. We then defined a constant multiplier C = exp(mean[log(subset of B) − log(subset of A)]) for each metabolic variable that was not a ratio. Lastly, the multiplier was applied to the subset from visit B to equalize the measurement scale. The procedure was applied first to the 2001 and 2007 visits, then to the 2007 and 2011 visits (24).

### Metabolomics and the Selection of Metabolic Features

A high-throughput NMR metabolomics platform was used. This particular quantitative methodology has been widely used in epidemiology and genetics over the last 10 years (with roughly a million samples analyzed) and data are currently available also in the UK Biobank (3, 27-29). The incorporated 143 molecular outputs from this metabolomics platform feature particle, total lipid, triglyceride, free cholesterol, esterified cholesterol, and phospholipid concentrations in multiple lipoprotein subclasses (very low-, intermediate-, low-, and high-density lipoprotein subclasses (30)), circulating apolipoprotein A-I and B concentrations, multiple clinical cholesterol (eg, remnant cholesterol) and triglyceride measures, lipoprotein particle sizes, albumin, phosphatidylcholine and sphingomyelin, various fatty acids (eg, omega-3 and omega-6 fatty acids; saturated as well as mono- and polyunsaturated fatty acids, linoleic and docosahexaenoic acid) and their ratios, and numerous low-molecular-weight metabolites, including amino acids (eg, branched-chain and aromatic amino acids), glycolysis related measures (eg, glucose, lactate and pyruvate), ketone bodies (acetate, acetoacetate, and 3-hydroxybutyrate), and a new inflammation marker GlycA—most of them in central pathways related to cardiometabolic health. In addition, 10 clinical traits were incorporated into the modeling: total, low- and high-density lipoprotein cholesterol, triglycerides, C-reactive protein, glucose, fasting insulin, body mass index, and systolic and diastolic blood pressure. These traits contained at most 5% of zero values (in order to prevent artifacts from excessive number of duplicates in the projection to latent structures [PLS]-modeling).

### Multivariate Prediction of Metabolic Age

Metabolic age was modeled by projection to latent structures (PLS). The input variables included 153 clinical and quantitative metabolic traits that were available in all the cohorts (24). Quantitative NMR metabolomics data, constituting multiple central pathways in cardiometabolic health, were used (as detailed above). This particular high-throughput methodology has been widely used in epidemiology over the last 10 years and data are currently available also in the UK Biobank (3, 4, 30). The training set included samples from the FINRISK 1997 and 2007 surveys (4666 men and 5042 women after quality control). Separate PLS models were trained for men and women. Before fitting the models, low-quality samples were filtered out and batch effects removed (31), the inputs were log-transformed if skewed, centered by mean and scaled by SD and any missing elements (<1.6%) were imputed by nearest-neighbor least-squares matching. The number of PLS components was optimized by screening the range between 2 and 50 by 7-fold cross-validation. The screening was replicated 50 times and the setting that produced the highest median Spearman correlation between predictions and observations was chosen for the final model (8 PLS components for both men and women). When the FINRISK-PLS was applied to the YFS, the inputs were standardized according to the means and SDs from the combined FINRISK surveys. The PLS coefficients are available in a Supplement table (32). All statistical analyses were conducted in the R environment version 3.6 and higher (https://www.R-project.org).

## Results

**Figure 2.**
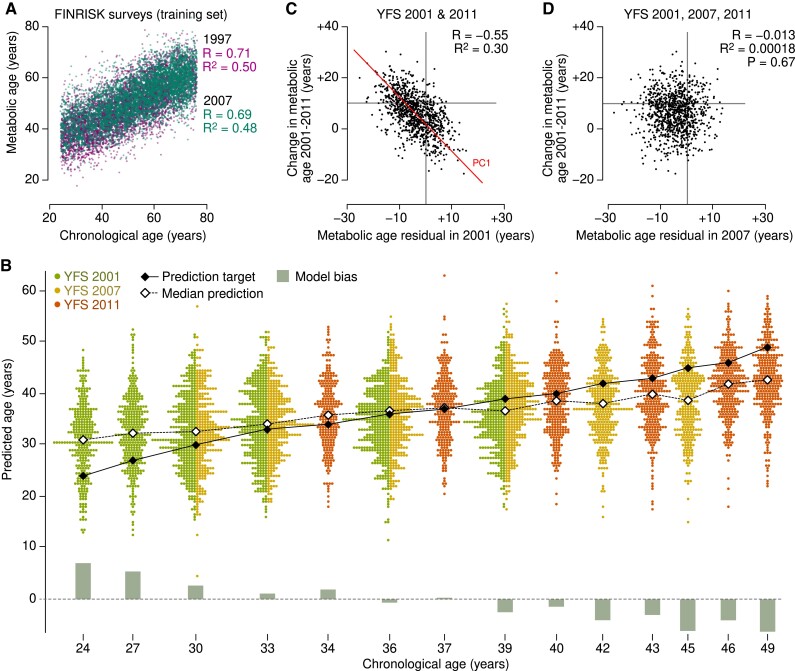
Metabolic age as a proxy for accelerated aging. (A) We used projections to latent structures (PLS) to predict an individual's chronological age from the cross-sectional FINRISK surveys. (B) The FINRISK-PLS model was applied to the YFS data for independent validation. Model bias (gray bars) was defined as the difference between the true chronological age (black filled diamonds) and the median of the model prediction (open diamonds) for a given peer group. (C) Accurate comparison of metabolic age residuals and longitudinal change is not possible with only 2 time points due to the negative correlation artifact caused by regression toward the mean. (D) Comparison between the predicted aging rate as indicated by metabolic age residuals in 2007 against the observed change in metabolic age as calculated between the 2001 and 2011 visits. Prediction performance is reported by Pearson correlation (R) and the horizontal gray lines indicate the +10-year expected change.

The statistical model of metabolic age was trained with the FINRISK survey data and then applied to the 3 YFS time points (Fig. 1C). Specifically, associations between the 153 clinical and metabolic measures and age were modeled by the PLS algorithm; this FINRISK-PLS model explained 50.0% of the variation in chronological age in the combined training set (Fig. 2A) and 18.4% of age variance in those FINRISK participants who were 49 years of age or younger (ie, the same age range as the YFS participants).

We applied the FINRISK-PLS model to the calibrated YFS data to investigate whether the trajectory of age-associated changes in metabolism could be predicted by the modeling of population surveys (Fig. 2B). The FINRISK-PLS model overestimated age for the youngest peer group (the mean residual was +7.0 years for the 24-year-olds) and underestimated age for the oldest peer group (−6.3 years for the 49-year-olds). Overall, the FINRISK-PLS explained 16.0% of the age variation in the YFS.

Lastly, we tested the hypothesis that a high predicted metabolic age compared with the chronological age of an individual is indicative of accelerated aging processes (Fig. 1A). First, we demonstrated the phenomenon of regression toward the mean by comparing the FINRISK-PLS residuals in YFS in 2001 against the observed differences between the model predictions in 2011 and 2001. As expected, a strong inverse correlation was observed (Fig. 2C). We then modified the design by switching to residuals from the 2007 visit in the middle to eliminate the confounding effect. We observed no correlation between the metabolic age residual in 2007 and the observed change in metabolic age during the follow-up period (*R^2^* = 0.018%, *P* = 0.67; Fig. 2D).

## Discussion

Multiple population-based cohorts were leveraged to investigate how aging manifests in systemic metabolism and whether modeling the metabolic age based on cross-sectional surveys provides information on an individual's metabolic trajectory (Fig. 1A). We found no evidence that cross-sectional modeling of aging provides information on how fast metabolic profiles change over time for an individual (Fig. 2D).

**Figure 3.**
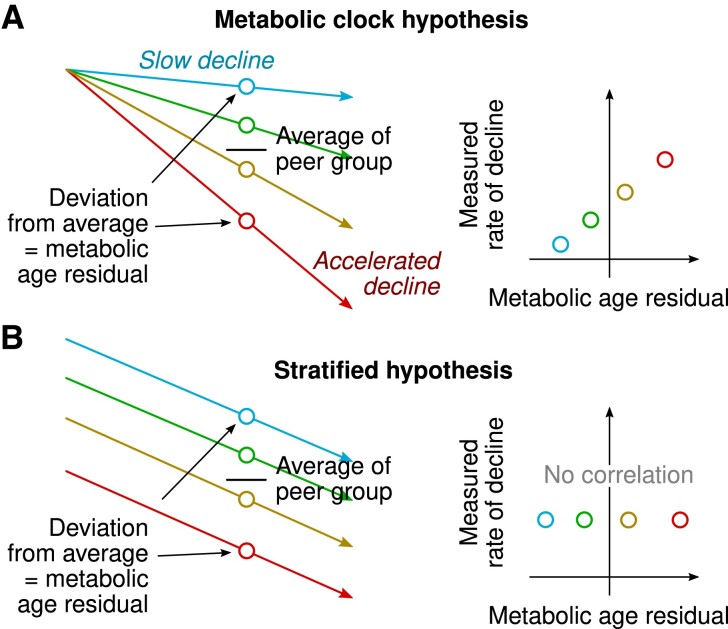
Hypothetical scenarios that generate different relationships between cross-sectional metabolic age residuals and longitudinally measured change in metabolic age. (A) Extrapolation of longitudinal trajectories from cross-sectional data under the molecular clock hypothesis where individuals start at the same level of health but decline at different rates. (B) A stratified model of aging where the fundamental rate of aging is constant but starting points are different. This model fits to our findings better than the metabolic clock model.

Experimental studies indicate that molecular processes related to aging are under genetic and epigenetic regulation, but it may be possible to slow down the molecular clock by caloric restriction and other interventions (1, 2, 17, 22). Demonstrating this phenomenon in humans is challenging, but the concept of metabolic age has been introduced as a proxy indicator of how far the clock has ticked (17, 22, 33, 34). Under the clock model, one could expect that a cross-sectional snapshot in the middle of the trajectory would reflect the divergent slopes that fan out from the starting point and that the snapshot could thus be used as a measure of accelerated aging (Fig. 3A). We could not detect the expected consequence of the molecular clock model of aging in young and middle-aged adults, as we could not establish a correlation between the metabolic age residuals and the observed change in metabolic age. As an alternative explanation, we speculate that the results arose from stratified aging where the rates of adult metabolic aging per se are not predictable by the current metabolic health status (Fig. 3B). In addition to statistical fluctuations, the metabolic age residuals are likely to reflect the genetic and environmental determinants of circulating metabolism and the interplay between the current degree of adiposity and energy metabolism.

These findings are consequential for the current aging research in metabolomics, as molecular aging signatures are regularly derived from cross-sectional data and interpreted as representations of individuals' biological ages. This approach appears to be mostly, if not entirely, misplaced.

## Data Availability

The datasets used in the current study are available from the cohorts through application process for researchers who meet the criteria for access to confidential data: https://thl.fi/en/web/thl-biobank/for-researchers/apply (FINRISK cohorts) and http://youngfinnsstudy.utu.fi (YFS). Regarding the YFS data, the Ethics committee has concluded that under applicable law, the data from this study cannot be stored in public repositories or otherwise made publicly available. The data controller may permit access on case-by-case basis for scientific research, not, however, to individual participant level data, but aggregated statistical data, which cannot be traced back to data for the individual participants.

## Abbreviations

NMR: nuclear magnetic resonance
YFS: Cardiovascular Risk in Young Finns Study
